# Characterization and Mapping of *retr04*, *retr05* and *retr06* Broad-Spectrum Resistances to Turnip Mosaic Virus in *Brassica juncea*, and the Development of Robust Methods for Utilizing Recalcitrant Genotyping Data

**DOI:** 10.3389/fpls.2021.787354

**Published:** 2022-01-12

**Authors:** Lawrence E. Bramham, Tongtong Wang, Erin E. Higgins, Isobel A. P. Parkin, Guy C. Barker, John A. Walsh

**Affiliations:** ^1^School of Life Sciences, University of Warwick, Wellesbourne Campus, Warwick, United Kingdom; ^2^Agriculture and Agri-Food Canada, Saskatoon, SK, Canada

**Keywords:** turnip mosaic virus, amphidiploid *Brassica juncea*, recessive TuMV resistance, linkage mapping, quantitative trait loci, genotyping by sequencing

## Abstract

Turnip mosaic virus (TuMV) induces disease in susceptible hosts, notably impacting cultivation of important crop species of the *Brassica* genus. Few effective plant viral disease management strategies exist with the majority of current approaches aiming to mitigate the virus indirectly through control of aphid vector species. Multiple sources of genetic resistance to TuMV have been identified previously, although the majority are strain-specific and have not been exploited commercially. Here, two *Brassica juncea* lines (TWBJ14 and TWBJ20) with resistance against important TuMV isolates (UK 1, vVIR24, CDN 1, and GBR 6) representing the most prevalent pathotypes of TuMV (1, 3, 4, and 4, respectively) and known to overcome other sources of resistance, have been identified and characterized. Genetic inheritance of both resistances was determined to be based on a recessive two-gene model. Using both single nucleotide polymorphism (SNP) array and genotyping by sequencing (GBS) methods, quantitative trait loci (QTL) analyses were performed using first backcross (BC_1_) genetic mapping populations segregating for TuMV resistance. Pairs of statistically significant TuMV resistance-associated QTLs with additive interactive effects were identified on chromosomes A03 and A06 for both TWBJ14 and TWBJ20 material. Complementation testing between these *B. juncea* lines indicated that one resistance-linked locus was shared. Following established resistance gene nomenclature for recessive TuMV resistance genes, these new resistance-associated loci have been termed *retr04* (chromosome A06, TWBJ14, and TWBJ20), *retr05* (A03, TWBJ14), and *retr06* (A03, TWBJ20). Genotyping by sequencing data investigated in parallel to robust SNP array data was highly suboptimal, with informative data not established for key BC_1_ parental samples. This necessitated careful consideration and the development of new methods for processing compromised data. Using reductive screening of potential markers according to allelic variation and the recombination observed across BC_1_ samples genotyped, compromised GBS data was rendered functional with near-equivalent QTL outputs to the SNP array data. The reductive screening strategy employed here offers an alternative to methods relying upon imputation or artificial correction of genotypic data and may prove effective for similar biparental QTL mapping studies.

## Introduction

Turnip mosaic virus (TuMV) belongs to the *Potyvirus* genus and causes significant economic losses through diminished harvest yield and produce quality of infected crops, particularly cultivated species of the *Brassica* genus (Walsh and Jenner, 2002). It can infect at least 318 plant species (Edwardson and Christie, 1991) and be rapidly spread by over 89 aphid vector species through the non-persistent transmission route (Walsh and Jenner, 2002). These factors contribute to its widespread distribution and economic impact, whilst also posing significant challenges for effective mitigation of associated disease. Historic management of TuMV has predominantly relied on insecticides for the non-specific control of aphid vector species, however, chemical control is considered increasingly unviable. This is partially due to resistances evolving across aphid populations against the mode-of-action(s) of key pesticides (Bass et al., 2014). Environmental concerns also exist as pesticide misuse/dependency continues to be a source of controversy with detrimental links suggested to non-target insect biodiversity (Lundin et al., 2015).

If implemented with careful consideration, the deployment of characterized natural genetic resistances *via* marker-assisted selection (MAS) is considered to be a reliable, more environmentally-friendly, economical, and socially acceptable strategy for mitigating viral disease (Shattuck, 1992; Kang et al., 2005). However, the identification, characterization and deployment of new resistance(s) can be challenging, often taking substantial time and effort, whilst depending upon robust genotyping methodologies.

*Brassica juncea* (AABB, 2*n* = 36) is an amphidiploid species originating from interspecific hybridization between the diploid species *Brassica rapa* (AA, 2*n* = 20) and *Brassica nigra* (BB, 2*n* = 16; Prakash et al., 2012). It is a cultivated crop species of global importance grown predominantly as a source of vegetable oil in India and Northwest China, with leafy vegetable, root vegetable, and stem/leaf crop morphotypes also being economically important (Huangfu et al., 2009). It constitutes a staple vegetable with high dietary consumption throughout Asia. China, specifically, represents a focus for considerable *B. juncea* diversity and where TuMV infection is particularly widespread, resulting in substantial yield losses (Huangfu et al., 2009). Impacts of TuMV are not limited to Asia, reductions of up to 85% of mature plant height and 84% seed yield have been recorded for *B. juncea* in Australian regions of cultivation with incidences of 25–100% suggested (Schwinghamer et al., 2014). The vast host range of TuMV enables reservoirs of the virus to exist both during and between growing seasons in and around field crops, often within wild weed species, where the presence of plants with TuMV symptoms has been recorded as high as 100% (Schwinghamer et al., 2014). Reliable estimates of yield losses in *B. juncea* due to TuMV infection are difficult to determine and generally inconsistent due to a range of factors including variations in local climate, the abundance of key aphid vector species, and the relative incidence of discrete TuMV strains with changing pathotypes/patterns of virulence.

Few large-scale investigations have been performed into screening diverse *B. juncea* germplasm against TuMV and even fewer where any level of TuMV resistance has been identified. Excluding those explored here, just two TuMV resistances have been reported in *B. juncea*. The first of these, TuMV Resistance in *B. juncea* No. 1 (*TuRBJU01*), identified by Nyalugwe et al. (2015) was incompletely dominant against TuMV isolates NSW-2, NSW-1 and WA-Ap, representing pathotypes 1, 7 and 8, respectively (Jenner and Walsh, 1996; Nyalugwe et al., 2016b). The necrotic response observed with *TuRBJU01* consisted of either systemic infection with some necrosis, or systemic hypersensitivity and plant death (Nyalugwe et al., 2015), so might not represent an agronomically attractive prospect. Identification of *TuRBJU01* involved challenging 69 *B. juncea* lines with the aforementioned TuMV isolates, following the earlier work of Kehoe et al. (2010) where an additional 44 *B. juncea* had been investigated. The *TuRBJU01* gene is yet to be to be cloned and/or mapped, and no resistance-associated molecular markers are currently published. Despite additional explorations of the underlying mechanism of *TuRBJU01*-based resistance (Nyalugwe et al., 2016a), there is no indication the resistance has been exploited. The second *B. juncea*-derived TuMV resistance, recessive TuMV resistance No. 3 (*retr03*), is a monogenic recessive source of resistance identified by Shopan et al. (2017) that was only effective against one of the TuMV isolates it has been tested against. Through bulked segregant analysis and sequencing of TuMV-resistant and susceptible *B. juncea* lines, *retr03* was indicated to be a variant of eukaryotic translation initiation factor 2B-beta (*eIF2Bβ*), which is critical for the initiation of eukaryotic protein synthesis and hijacked by TuMV during infection (Bogorad et al., 2014; Shopan et al., 2017). *TuRBJU01* and *retr03* were the only sources of TuMV resistance available for developing TuMV-resistant *B. juncea* cultivars through introgression of the relevant gene(s) *via* MAS. However, as *TuRBJU01* results in plant death and *retr03* has been overcome by a number of TuMV isolates, these resistances are unlikely to be durable.

Genetic mapping of valuable traits can present many challenges. Lack of sufficient recombination captured across a mapping population, failure to genotype highly informative samples, apparent low overall genotyping quality and additional considerations such as polyploidy and the absence of a truly representative species reference genome are a small subset of issues which may be faced (Jones et al., 2009; Semagn et al., 2010; Nadeem et al., 2018). The quantitative trait loci (QTL) analyses performed here necessitated original approaches for effective data handling to overcome issues including these.

Projections suggest that pathogens and pests affecting global crops will move away from the equator with increasing temperature (Bebber et al., 2013). Due to brassica crops being cultivated worldwide, observed trends in climate change are very likely to disrupt the distribution and abundance of key aphid vector species across regions of cultivation and conceivably also impact associated TuMV strain variation. Consequently, additional sources of resistance remain of high importance, particularly those with broader spectra of resistance and/or efficacy against the most common TuMV pathotypes, 1, 3 and 4 (Jenner and Walsh, 1996).

Two new TuMV resistance sources effective against key TuMV isolates/pathotypes, identified in *B. juncea* germplasm, are presented here. Robust QTL analyses were performed and three new recessive genes, *retr04*, *retr05* and *retr06*, providing broad spectrum resistance are described, along with methods for handling recalcitrant genotyping data.

## Materials and Methods

### Plant Material, Growth Conditions, and Crossing

A total of 33 lines of *B. juncea* spanning various genetic and geographic origins were evaluated for resistance against TuMV isolate UK 1 (Table 1). Two of these lines, TWBJ14 and TWBJ20, were taken forward and developed into two first backcross (BC_1_) mapping populations shown to segregate for resistance to TuMV UK 1. Both TWBJ14 and TWBJ20 material were of the root morphotype and originated from China. The TuMV-susceptible *B. juncea* line 060DH17 line was used as the maternal parental line during initial crosses with TWBJ14 and TWBJ20 sources of TuMV resistance to produce the F_1_ populations. This line is TuMV UK 1-susceptible, a doubled haploid and therefore homozygous across all genomic loci. Routine practices were employed for all plant crosses and for establishing an informative BC_1_ population following phenotyping of relevant self and filial populations. The TWBJ14 and TWBJ20 BC_1_ mapping populations were developed by crossing F_1_ (060DH17 ♀ × TuMV-resistant plants ♂) UK 1-susceptible plants (♀) to TuMV UK 1-resistant S_1_ plants (♂) that had been generated from the original resistant plants of each line used for initial crossing.

**Table 1:** Geographical origins, morphotypes, and response of *B. juncea* plant lines tested for resistance to TuMV following mechanical inoculation with TuMV UK 1.

*Brassica juncea* plant line	Geographical origin	Morphotype	No. plants infected/no. tested	Phenotype^*1^	Systemic infection
TWBJ01	Malaysia	Leaf	10/10	+	Yes
TWBJ02	Bhutan	Oilseed	10/10	+	Yes
TWBJ03^*2^	Bhutan	Oilseed	2/10	+_N_/0	Yes/No
TWBJ04^*2^	Bhutan	Oilseed	7/10	+_N_/R	Yes/No
TWBJ05	Zimbabwe	Unconfirmed	10/10	+_N_	Yes
TWBJ06^*2^	Zimbabwe	Unconfirmed	5/10	+/0	Yes/No
TWBJ07	Zimbabwe	Unconfirmed	10/10	+	Yes
TWBJ08	Zimbabwe	Unconfirmed	9/9	+_N_	Yes
TWBJ09	Zimbabwe	Unconfirmed	10/10	+	Yes
TWBJ10	Zimbabwe	Unconfirmed	10/10	+	Yes
TWBJ11	Zimbabwe	Unconfirmed	10/10	+	Yes
TWBJ12	Zimbabwe	Unconfirmed	10/10	+_N_	Yes
TWBJ13	Zimbabwe	Unconfirmed	10/10	+	Yes
TWBJ14^*2^	China	Root	0/10	0	No
TWBJ15^*2^	China	Leaf	0/3	R	No
TWBJ16	China	Leaf	10/10	+	Yes
TWBJ17	China	Stem	10/10	+_N_	Yes
TWBJ18^*2^	China	Stem	8/10	+/R	Yes/No
TWBJ19	China	Leaf	10/10	+	Yes
TWBJ20^*2^	China	Root	1/10	+/0	Yes/No
TWBJ21	Bhutan	Oilseed	10/10	+	Yes
TWBJ22	Hong Kong	Leaf	10/10	+	Yes
TWBJ23^*2^	China	Root	0/10	0	No
TWBJ24	Uruguay	Unconfirmed	7/7	+_N_	Yes
TWBJ25	Japan	Leaf	10/10	+	Yes
TWBJ26	Japan	Leaf	10/10	+	Yes
TWBJ28	Unconfirmed	Unconfirmed	10/10	+_N_	Yes
TWBJ29	China	Leaf	10/10	+	Yes
TWBJ30	Japan	Oilseed	10/10	+	Yes
TWBJ31	India	Oilseed	10/10	+_N_	Yes
TWBJ32	India	Oilseed	10/10	+_N_	Yes
TWBJ33	U.K.	Leaf	10/10	+	Yes
TWBJ34	India	Oilseed	9/9	+_N_	Yes

*1 Phenotypes were scored according to the system of Jenner and Walsh (1996). 0, resistance with no TuMV-associated symptoms and no TuMV detected by ELISA; R, resistance where infection was limited to inoculated leaves with no systemic spread; +, systemic TuMV infection or +_N_, systemic infection with necrosis. TuMV detection using plate-trapped antigen ELISA (PTA-ELISA) confirmed the resistance/susceptibility status of all plants.

*2 Eight lines demonstrated some level of resistance.

Resistance complementation testing was performed to elucidate whether resistance-associated genes were shared between TWBJ14 and TWBJ20 material through selective crossing and phenotyping of progeny with TuMV isolate GBR 6. Material used for complementation testing were F_2_ populations generated by selfing the progeny of reciprocal crosses between TuMV-resistant TWBJ14 and TWBJ20 S_2_ plants.

*Brassica rapa* ssp. *perviridis* cv. Tendergreen (Mustard-Spinach; TGM) is a highly susceptible host of TuMV and was used to maintain TuMV isolates through serial inoculations, and as a TuMV-susceptible experimental control to validate viral inocula during all testing. A plant line from the pathotyping system developed by Jenner and Walsh (1996), *Brassica napus* line R4 possessing *TuRB01*-based resistance to TuMV along with back-inoculation and reinoculation strategies were employed to confirm the authenticity and stability of TuMV isolates during phenotyping.

All experimental plant material was grown from seed within an insect-proof glasshouse maintained at 18 ± 2°C. Natural light levels were monitored and supplementary lights used in daytime when levels fell below 600 W/m^2^. Seeds were planted in FP7 pots (7 cm square) containing Pot and Bedding M2 compost (Levington; medium grade sphagnum moss peat, pH 5.3–6.0). Plants were subsequently transferred to successively larger pots (FP9 and FP11; 9 and 11 cm square, respectively) containing John Innes No. 2 potting-on compost (Erin; loam-based compost with added fertilizer, pH ~6.5) when normal plant growth was hindered by available nutrition.

### TuMV Isolates and Resistance Phenotyping

Four TuMV isolates, UK 1, v35Tunos +5570 A > G (vVIR24), CDN 1 and GBR 6 were employed within this study. They represent the most common TuMV pathotypes, 1, 3, 4 and 4, respectively (Jenner and Walsh, 1996; Jenner et al., 2000). TuMV UK 1 was obtained from swede in England (Tomlinson and Ward, 1978; Walsh, 1989) and is unable to overcome the TuMV resistance gene *TuRB01* (Walsh et al., 1999). TuMV vVIR24 is an engineered mutant of UK 1 containing a single nucleotide mutation, conferring the ability to overcome *TuRB01*-based resistance (Jenner et al., 2000). TuMV CDN 1 was obtained from rutabaga in Canada (Walsh, 1989). It can overcome *TuRB01* and at least three additional sources of resistance to UK 1 (Jenner et al., 2002b). Like CDN 1, TuMV GBR 6 is a pathotype 4 isolate. It has, however, been shown to overcome *TuRB03*-based TuMV resistance (Hughes et al., 2003); limited sources of resistance have been identified to this isolate.

As described by Walsh (1989), TuMV challenges were performed by mechanical inoculation of test plants at the 2–5 true leaf growth stage. TuMV-infected TGM leaf tissue was homogenized in cold inoculation buffer (K_2_HPO_4_, 10 g/L; Na_2_SO_3_, 1 g/L) using a sterile pestle and mortar. The resulting mixture was then abraded using sterile muslin cloth onto the adaxial surface of target plant leaves dusted with fine carborundum grit (~36 μm; ThermoFisher Scientific, United Kingdom). The abrasion and lysis of cells provided TuMV access to epidermal/parenchymatous cells in a controllable and uniform manner. The newest inoculated leaf on each plant was marked in order to discriminate between TuMV-inoculated and new, uninoculated leaves; this facilitated assessment of systemic viral spread. Uninfected TGM leaf tissue was used to mock-challenge experimental control plants.

**Figure 1:**
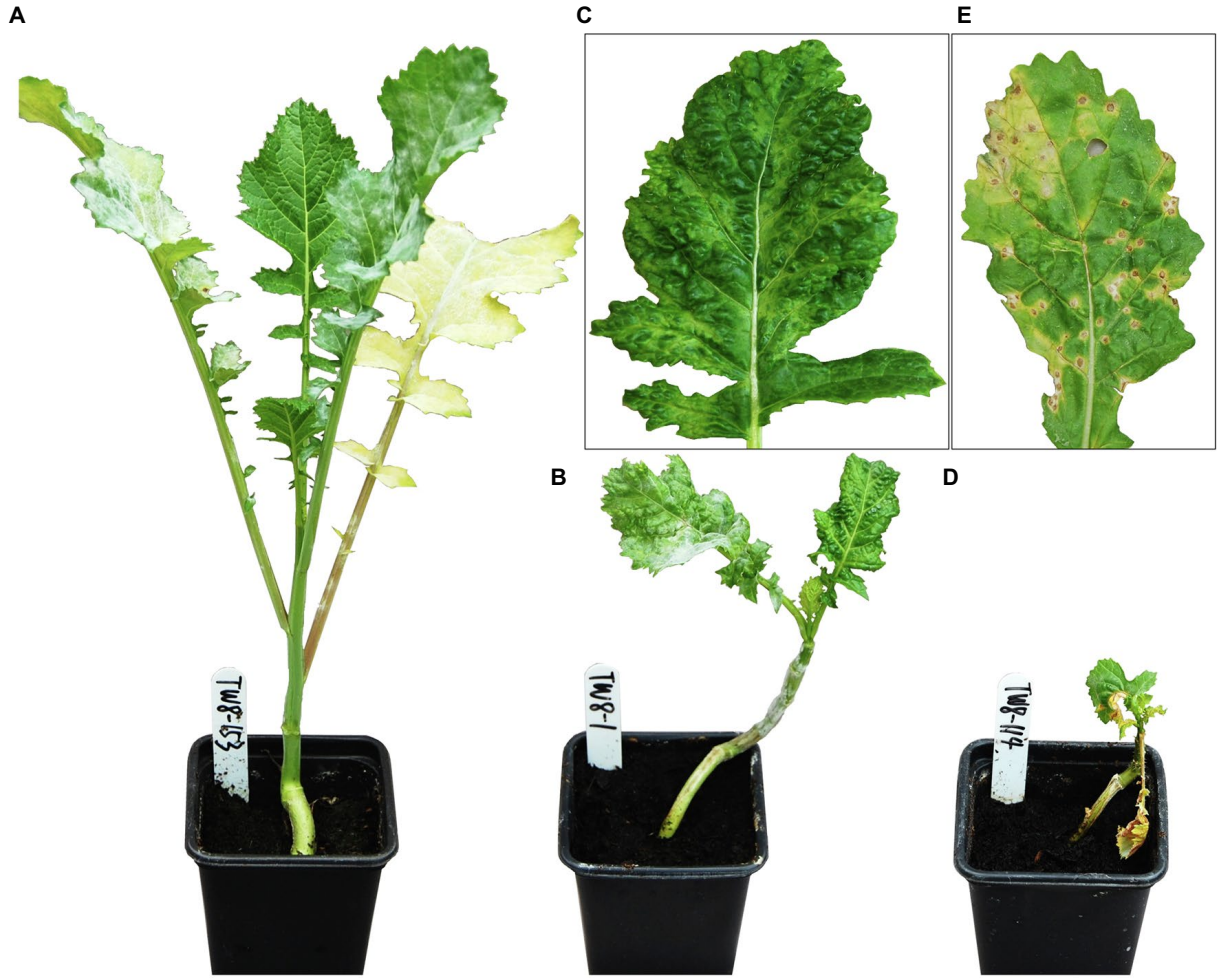
Distinct visual phenotypes observed across TWBJ14 *Brassica juncea* BC_1_ population four weeks post-challenge with turnip mosaic virus (TuMV) isolate UK 1. **(A)** No infection (0), plants appeared resistant; **(B)** Systemic infection (+). **(C)** Enlarged view of single leaf from plant **B**. **(D)** Systemic infection with necrosis (+_N_); **(E)** Enlarged view of single leaf from plant **D**. Phenotypes are representative of those observed across all plant generations tested.

Turnip mosaic virus-associated symptoms were scored weekly through visual inspection for four weeks post-inoculation, as described by Jenner and Walsh (1996). Qualitative visual phenotypes comprised of, “0,” resistance with no TuMV-associated symptoms, “R,” resistance where infection was limited to inoculated leaves with no systemic infection, “+,” systemic TuMV infection or +_N_, systemic infection with necrosis (Figure 1). TuMV infection and titer were confirmed following visual assessment, using an indirect plate-trapped antigen ELISA (PTA-ELISA), as described by Walsh et al. (2002). Equal sized unchallenged and challenged leaves from test plants were harvested, macerated and diluted 1:1 with 0.05 M sodium carbonate coating buffer (Na_2_CO_3_, 1.6 g/L; NaHCO_3_, 3 g/L). Test samples and appropriate TuMV-infected and uninfected control material were pipetted into predefined duplicate wells of a 96-well microtiter plate then incubated for 12 h at 4°C. Standard PTA-ELISA methods were followed with the first/detection antibody EMA67, a mouse monoclonal antibody, used at a concentration of 1/500. EMA67 has been shown capable of recognizing all tested isolates of TuMV by targeting a conserved TuMV coat protein epitope (Jenner et al., 1999). The second/visualization antibody, a goat anti-mouse IgG conjugated to alkaline phosphatase (Sigma-Aldrich, United Kingdom), was used at a concentration of 1/2,000. After all unbound antibodies were removed, plates were incubated at room temperature with p-nitrophenyl phosphate substrate solution (1 mg/ml in 0.1 M diethanolamine, pH 9.8). The reaction between the alkaline phosphatase conjugated to the detection antibody and this substrate produced a color change, which was used to indirectly quantify relative viral titer within samples.

### Genotyping Methods and Quantitative Trait Loci Analyses

Genotyping of mapping populations was performed using two methods. A total of 218 TWBJ14 BC_1_ plants (52 resistant/0; 105 susceptible/+; 61 susceptible/+_N_), 158 TWBJ20 BC_1_ (22 resistant/0; 136/+_N_) and the four parental plants used for the two initial F_1_ crosses were genotyped using a 90 K single nucleotide polymorphism (SNP) array containing informative polymorphisms spanning all “A,” “B,” and “C” *Brassica* genomes (unpublished, SNP array under development and provided by the Parkin group, Agriculture and Agri-Food Canada; AAFC). This was complemented by genotyping by sequencing (GBS) on 102 TWBJ14 BC_1_ (41 resistant/0; 31 susceptible/+; 30 susceptible/+_N_) plant samples performed using the method of Poland et al. (2012). DNA was normalized to 20 ng/μl and 200 ng digested with PstI and MspI restriction enzymes at 37°C for 2 h. Adapters were then ligated to the digested DNA fragments using T4 DNA ligase for 2 h at 22°C. The reaction was inactivated, and samples pooled in two sets (96 samples in one set with those remaining processed in parallel alongside other samples, totaling 96). After pooling, the two libraries were amplified with a short extension time of 30 s and then purified using a QIAquick PCR Purification Kit (Qiagen). An Agilent Bioanalyzer was used to confirm fragment size and quality of the libraries. Libraries were then quantified using the Kapa library quantification kit (Roche) and sequenced on an Illumina HiScanSQ (100 bp paired-end reads). An existing pipeline was used to demultiplex sequencing reads. Trimmomatic (Bolger et al., 2014) was used to trim the adapters, and remove short reads and poor-quality data. Leading and trailing bases with quality below 15 and reads shorter than 55 bp were removed. The trimmed sequence reads were aligned to a *B. juncea* reference genome under development (IAP Parkin, AAFC, unpublished) using Bowtie2 (Langmead and Salzberg, 2012) with “-local” and “-sensitive” parameters used and “-score-min” of “L, 0, 0.8”. A custom Perl script was used to extract the best unique hits and the resulting BAM files were used for variant calling. UnifiedGenotyper with standard parameters from the Genome Analysis Toolkit (McKenna et al., 2010) was used to call polymorphic loci.

In addition to mapping resistance, a necrotic phenotype observed in the TWBJ14 BC_1_ population was mapped using the 90K SNP array. The analysis was based solely on TuMV-susceptible plants, as it was not possible to determine whether resistant plants possessed the QTL associated with the necrotic phenotype induced by TuMV infection.

All QTL statistical analyses were performed using R v3.4.4. The add-on package “R/qtl” (Broman et al., 2003; Arends et al., 2010) was used to filter, model, and visualize genotypic data using inbuilt algorithms and according to established QTL mapping methods (Haley and Knott, 1992; Jones et al., 2009; Semagn et al., 2010; Xu et al., 2017). Prior to linkage group generation, a range of custom filters were successively applied to identify reliable marker subsets without the need for imputation or reliance on traditional measures of marker quality, such as sequencing read depth (Supplementary Tables 1 and 2). Pairwise recombination fraction (RF) values were calculated between all final marker subsets and a logarithm of the odds (LOD) score calculated for each pair based on a likelihood test of RF = 0.5. Linkage groups were inferred using appropriate RF and LOD score values adjusted to a stage where an appropriate number of linkage groups/chromosomes were generated. Ordering of markers within linkage groups was performed independently of reference genome position-associated physical loci. All possible orders within a successively increasing subset of markers were considered and applied where the number of obligate recombination events were reduced. After all markers had been ordered, linkage maps were finalized by calculating inter-marker distances based on RF using the Haldane mapping function, assuming no meiotic crossover interference. In addition to estimating inter-marker distances, the Haldane mapping algorithm was used to calculate an associated LOD score based on a test of how dependable calculated distances were. Using a selection of potential/assumed genotyping error rates, this LOD score was used to indicate the likely true error of genotyping present within final marker subsets.

Due to observed phenotypes being qualitative (i.e., distinct TuMV resistance against susceptibility), appropriate nonparametric algorithms were used for all QTL analyses. Multiple QTL modeling was first used to predict the most likely number of QTLs and any interaction between them using a stepwise forward/backward search algorithm, assuming a maximum of four distinct QTLs succeeded by permutation-based testing of the suggested QTL model (1,000 permutations, *α* < 0.05). Suitable Haley-Knott, multiple imputation-based, and expectation-maximization QTL interval mapping (IM) algorithms were then used to identify marker-phenotypic variance associations. Algorithm-specific statistical likelihood LOD scores indicating the probability of any marker being associated with the phenotype undergoing mapping were determined for all markers. To complement QTL modeling, cofactors were also applied through standard composite interval mapping (CIM) techniques to infer whether multiple QTLs with minor effects were likely present (Jansen, 1993; Zeng, 1994). Genome-wide LOD significance thresholds were determined for all QTL mapping algorithms through 10,000 permutations of randomized phenotypic data and *α* < 0.05. QTL CIs of a 1.5 LOD drop from the peak marker-associated LOD score were identified, and QTL intervals defined by extending this interval to adjacent flanking markers of each linkage map.

## Results

### TuMV Resistance Phenotyping

From the 33 *B. juncea* lines screened against TuMV isolate UK 1, eight were identified with some level of resistance (Table 1). Two S_2_ populations produced by selfing both TuMV-resistant TWBJ14 S_1_ and TWBJ20 S_1_ plants exhibited uniform resistance when challenged with TuMV isolates UK 1, vVIR24 and CDN 1 (Table 2). This indicated that both plant lines possessed broad-spectrum resistance against isolates representing the most prevalent pathotypes (1, 3 and 4) of TuMV. Phenotyping of F_1_ populations (060DH17 ♀ × TuMV-resistant plants ♂) for resistance to TuMV UK 1 suggested that both sources of resistance were recessive (Table 3). Subsequent phenotyping of both TWBJ14 and TWBJ20 F_2_ and BC_1_ populations further reinforced this; the observed patterns of segregation between TuMV resistance and susceptibility did not differ significantly according to chi-square (*χ*^2^) analyses from expected ratios based on a model of two recessive genes (TWBJ14 F_2_ *χ*^2^ = 0.094, TWBJ14 BC_1_ *χ*^2^ = 0.150, TWBJ20 BC_1_ *χ*^2^ = 2.38 < *χ*^2^_0.05_ = 3.84; Table 4). TuMV-susceptible experimental control TGM plants challenged in all of these experiments were clearly infected with TuMV. Throughout all phenotyping experiments, there was also no indication that TuMV isolates behaved inconsistently when inoculated to the TuMV pathotype 1-resistant R4 plant line.

**Table 2:** Response of *B. juncea* S_2_ plant lines and *Brassica napus* line R4 following mechanical inoculation with turnip mosaic virus isolates UK 1, vVIR24 and CDN 1.

Plant line (population)	TuMV isolate (pathotype)					
	UK 1 (1)		vVir24 (3)		CDN 1 (4)	
		No. plants infected/no. tested	Phenotype^*^	No. plants infected/no. tested	Phenotype^*^	No. plants infected/no. tested	Phenotype^*^
TWBJ14 S_2_	0/9	0	0/9	0	0/9	0
TWBJ20 S_2_	0/9	0	0/9	0	0/9	0
R4	0/2	0	2/2	+_N_	2/2	+

* Phenotypes were scored according to the system of Jenner and Walsh (1996). 0, resistance with no TuMV-associated symptoms and no TuMV detected by ELISA, or +_N_, systemic TuMV infection with necrosis. TuMV detection using PTA-ELISA confirmed the resistance/susceptibility status of all plants.

**Table 3:** Response of S_1_ and F_1_ populations of *B. juncea* plant lines following mechanical inoculation with turnip mosaic virus isolate UK 1.

Plant line (population)	No. plants infected/no. tested	Phenotype^*^
TWBJ14 (S_1_)	0/28	0
TWBJ14 (F_1_)	5/5	+_N_
TWBJ20 (S_1_)	0/28	0
TWBJ20 (F_1_)	15/15	+_N_

* Phenotypes were scored according to the system of Jenner and Walsh (1996).

0, resistance with no TuMV-associated symptoms and no TuMV detected by ELISA, or +_N_, systemic TuMV infection with necrosis. TuMV detection using plate-trapped antigen PTA-ELISA confirmed the resistance/susceptibility status of all plants.

**Table 4:** Response of S_2_ and F_2_ populations of *B. juncea* plant lines following mechanical inoculation with turnip mosaic virus isolate UK 1.

Plant line (population)	No. plants (phenotype^*1^)			Predicted ratio for TuMV resistance:susceptibility^*2^	Goodness of fit/*χ*^2^ value^*3^
	Resistant	Susceptible			
	(0)	(+)	(+_N_)		
TWBJ14 (S_2_)	10	0	0		
TWBJ14 (F_2_)	9	81	69	1:15	0.094
TWBJ14 (BC_1_)	53	109	60	1:3	0.150
TWBJ20 (S_2_)	20	0	0		
TWBJ20 (BC_1_)	41	0	161	1:3	2.38

*1 Phenotypes were scored according to the system of Jenner and Walsh (1996). 0, resistance with no TuMV-associated symptoms and no TuMV detected by ELISA; +, systemic TuMV infection or +_N_, systemic infection with necrosis. TuMV detection using plate-trapped antigen PTA-ELISA confirmed the resistance/susceptibility status of all plants.

*2 Predicted ratio for each plant population presented as the number of TuMV-resistant plants: TuMV-susceptible plants (0: +/+_N_), according to a two recessive gene model.

*3 Goodness of fit based on chi-square (*χ*^2^) method; with one degree of freedom: *χ*^2^_0.05_ = 3.84.

Associated *p*-values were > 0.05 in all instances.

### Genotyping by Sequencing and 90K SNP Array Outputs for TWBJ14 and TWBJ20 BC_1_ Populations

Inspection of 90K SNP array-derived genotypic data suggested raw data for both TWBJ14 and TWBJ20 BC_1_ mapping populations was of high quality. A total of 11,732 marker loci were successfully genotyped with relatively few failing to be genotyped across less than 90% of plants from each of the BC_1_ populations assessed (2.1 and 3.4%, respectively, for TWBJ14 and TWBJ20). The vast majority of markers provided data across more than 99% of the BC_1_ samples tested (95.4 and 71.5% of TWBJ14 and TWBJ20, respectively). However, these marker loci were not strictly polymorphic and/or necessarily informative.

In contrast to the 90K SNP array data, all TWBJ14 GBS marker loci were identified based on a minimum of one sample being polymorphic to any other sample of the same BC_1_ population assessed, so theoretically should have provided a higher abundance of informative and germplasm-specific data. Substantially more loci were immediately identified as potentially useful using this methodology and due to GBS not relying on pre-determined marker loci of interest. A total of 47,845 SNPs or insertion/deletion events (InDels) were identified. Irrespective of these having the potential of being informative, further inspection of GBS data suggested near-complete absence of any useful data. Of all GBS-derived marker loci, 85.1% failed to be genotyped across more than 10% of plants tested; there were no notable patterns linking any discrete sample(s) or marker loci to genotyping failure. Of the remaining genotyped loci, where more than 90% of BC_1_ samples provided some level of data, sequencing read depth was highly variable. Due to the original parents of the TWBJ14 BC_1_ population not being available for processing by GBS, prospective markers could not be filtered based on observations of appropriate allelic variation or hetero/homozygosity of absent parental genotypic data. Approximately half of the TWBJ14 BC_1_ samples genotyped *via* the 90K SNP array (*n* = 218) were assessed using GBS (*n* = 102), further confounding issues with the efficacy of any recombination-based marker filtering strategies. These were limited due to inherently fewer recombination events being captured.

### Screening of Genotypic Data and Genetic Linkage Map Construction

Successive filtering of marker loci proved highly effective when based on a combination of initial qualitative parameters (e.g., for 90K SNP array data, removal of markers where the doubled haploid parental line 060DH17 was genotyped as heterozygous) followed by iterative and optimizable measures, such as removing genotypic data generated across <75% of samples (Supplementary Tables 1 and 2). Applying this reductive approach to filter prospective markers, the 90K SNP array data generated a final subset of 1,663 and 3,158 markers, respectively from the TWBJ14 and TWBJ20 BC_1_ populations. Recombination captured across each BC_1_ population was identified as a limiting factor. The removal of redundant markers, where calculated RF was near-identical and which would consequently provide no additional linkage map resolution, reduced the final subset of markers to 1,064 for TWBJ14 and 966 markers for TWBJ20 (Supplementary Table 1). Assumed genotyping errors employed with the Haldane linkage mapping function suggested an impactful error rate of 0.00% across both these final subsets of 90K SNP array data.

Comprehensive exploration of filtering strategies based on sequencing read depth were unsuccessful in producing a TWBJ14 GBS marker subset capable of allowing linkage map production based exclusively on recombination. The use of imputation-based methods aimed at remediating the impact of genotyping failure also proved ineffective when implemented at any stage of marker filtering. Ultimately, the lack of genotyping data from the original TWBJ14 source of resistance and parents of the BC_1_ population also proved extremely adverse. The identification of heterozygosity/recombination events across BC_1_ plants was still assessable, based on observations of consistent genotyping of discrete alleles across BC_1_ samples. Using this approach and similar reductive filtering as for the 90K SNP array data, a subset of robust markers was successfully identified from TWBJ14 GBS data (Supplementary Table 2). This final collection of markers was approximately 1% of the SNPs/InDels initially identified, totaling 481 unique loci. It is of note that this subset included informative markers which were otherwise discarded when implementing filtering strategies based on sequencing read depth. A small but potentially significant genotyping error rate of 0.55% was predicted when the final marker subset was assigned linkage groups and markers artificially ordered according to the *B. juncea* reference assembly.

**Figure 2:**
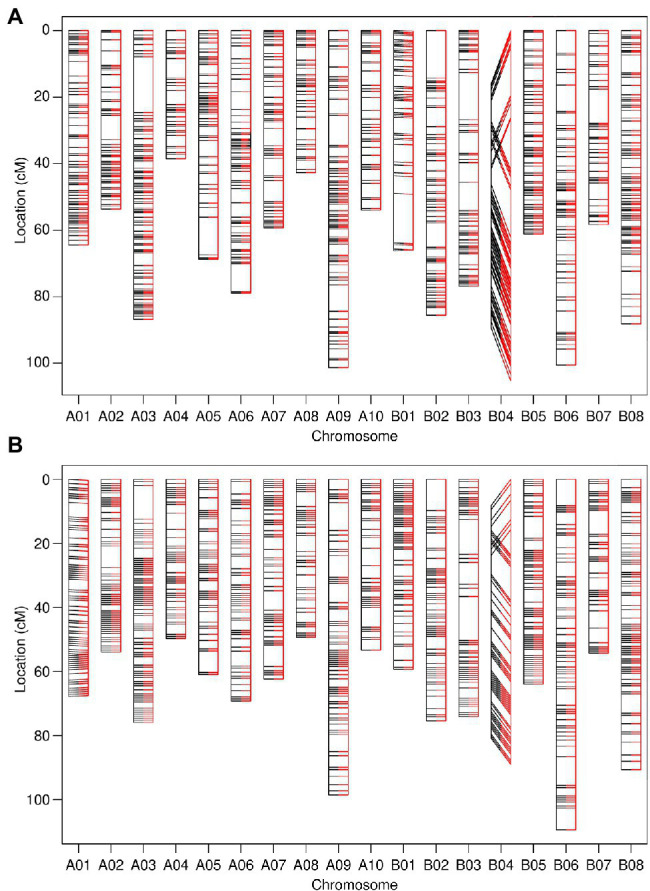
Genetic linkage maps constructed from 90K single nucleotide polymorphism (SNP) array genotyping of the TWBJ14 **(A)** and TWBJ20 **(B)** BC_1_ TuMV resistance-mapping populations. Marker loci ordered exclusively *via* calculated pairwise recombination fraction (black, left) and comparative order produced based on physical/*B. juncea* reference genome loci (red, right) are also presented. All inter-marker distances calculated based on recombination fraction. Connecting lines indicate the relative loci of identical markers within each linkage map.

Assignment of linkage groups and genetic map construction were performed during marker screening and employed as a method for the removal of unreliable genotypic data. The recombination fraction and associated LOD score calculated between approximately 7,100 filtered 90K SNP array markers from both *B. juncea* TWBJ14 and TWBJ20 BC_1_ populations allowed assignment of markers into linkage groups based on a maximum RF of 0.15 and minimum LOD of 8 (Supplementary Table 1). This set of values retained the maximum number of informative marker loci possible, while remaining stringent. The high quality of 90K SNP array genotyping enabled marker loci to be assigned to 18 linkage groups, representing the 18 chromosomes of *B. juncea*, with few markers remaining unassigned. A single marker from TWBJ14 and 12 from TWBJ20 SNP array datasets were removed during linkage group assignment. Ordering 90K SNP array markers exclusively according to recombination fraction generated linkage maps with near-identical orders to those where markers were artificially ordered according to physical *B. juncea* reference genome loci (Figure 2). The only exception to this was within chromosome B04 where a partial chromosome inversion event was suggested for both TWBJ14 and TWBJ20 germplasm. Distribution of markers and overall marker density was consistent across final genetic linkage maps generated from filtered TWBJ14 and TWBJ20 BC_1_ 90K SNP array marker data. The mean number of markers assigned to each chromosome for the TWBJ14 BC_1_ population was 59.1 ± 3.7 (SEM), and 53.7 ± 2.7 (SEM) for TWBJ20 with no apparent gaps in genome-wide coverage (Figure 2).

**Figure 3:**
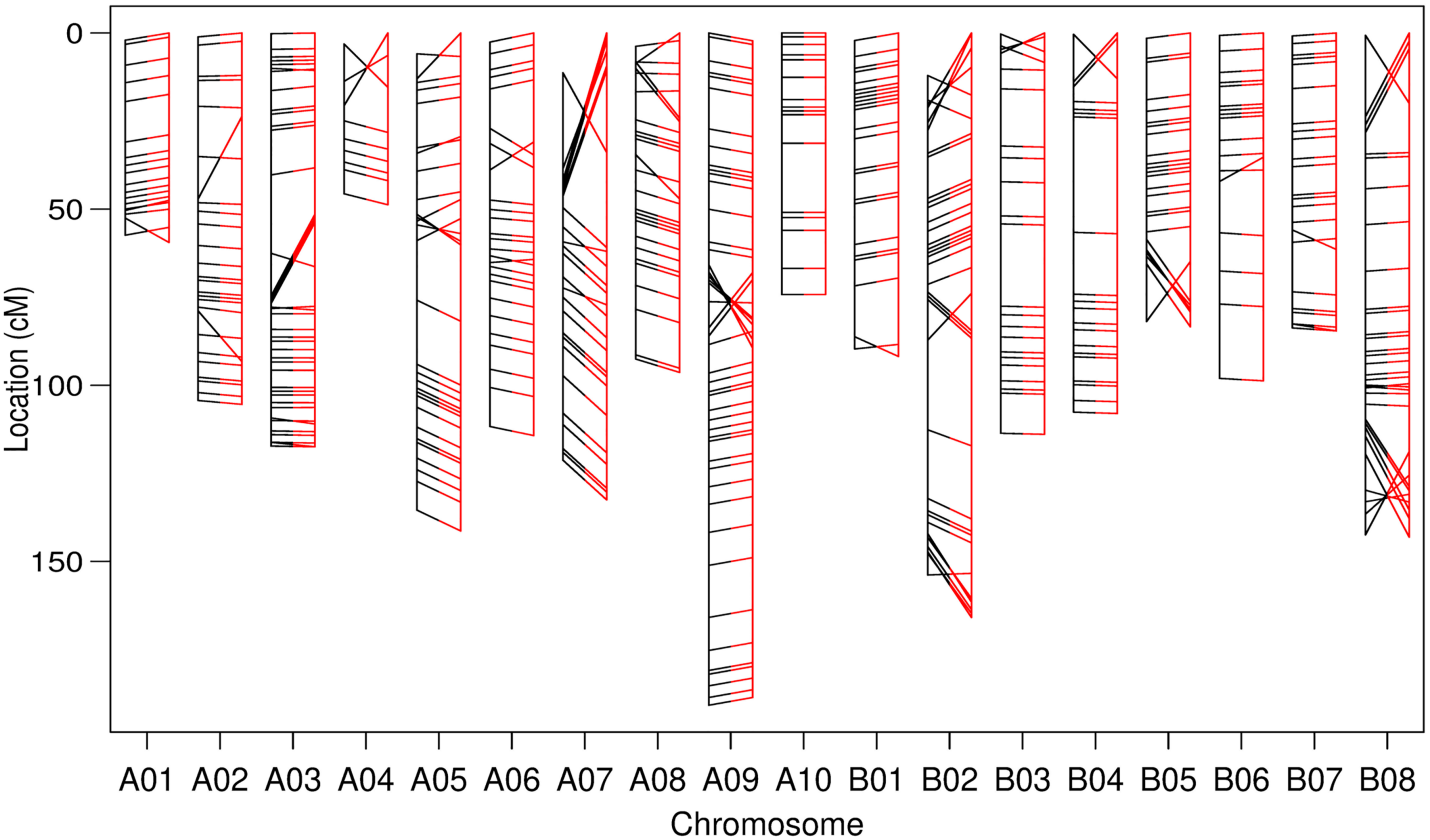
Genetic linkage map constructed from filtered genotyping by sequencing (GBS) data from the TWBJ14 BC_1_ turnip mosaic virus resistance-mapping population. Marker loci ordered exclusively *via* calculated pairwise recombination fraction (black, left) and comparative order produced based on physical/*B. juncea* reference genome loci (red, right) are also presented. All inter-marker distances calculated based on recombination fraction. Connecting lines indicate the relative loci of identical markers within each linkage map.

A linkage map was also constructed based exclusively on pairwise recombination calculated between the most reliable 481 TWBJ14 BC_1_ GBS markers remaining after excluding those presenting non-identical recombination. Compared to the physical order of markers on the *B. juncea* reference genome assembly, this linkage map could not be considered strictly reliable (Figure 3). Incorrect marker ordering occurred across many linkage groups, although, the majority of marker loci ordered by linkage were equivalent to orders predicted by physical reference assembly positions. Despite the suggestion from preliminary analyses of TWBJ14 GBS data that no viable data was present, a potentially informative linkage map was produced using considered screening of highly recalcitrant genotyping data.

### QTL Resistance Mapping and Interval Calculation

#### TWBJ14 90K SNP Array

**Figure 4:**
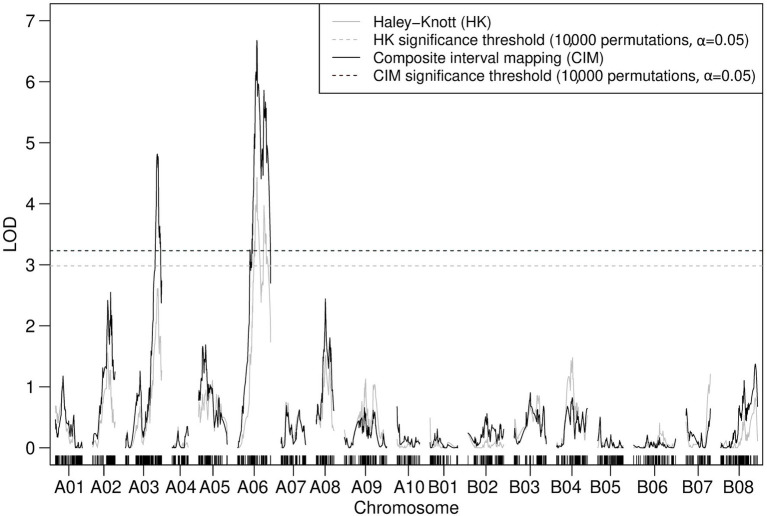
Logarithm of the odds (LOD) profile of nonparametric composite interval mapping (CIM) with two cofactors (assigned to the peak LOD scores for A03 and A06 determined by interval mapping; IM) performed on the TWBJ14 *B. juncea* BC_1_ TuMV resistance mapping population using 90K SNP array data. The TuMV resistance quantitative trait loci (QTLs) on A03 (*retr05*) and on A06 (*retr04*) passed the threshold (black-dashed line) for significance (LOD 3.23; based on an alpha significance value of 0.05 and 10,000 permutations). The results and significance threshold of nonparametric IM, assuming a single QTL model, using the Haley-Knot algorithm (gray) are also presented.

Iterative forward and backward consideration of the addition of TWBJ14 90K SNP array linkage map markers into a model attempting to account for observed phenotypic variation indicated that a model of two loci for TuMV resistance, one on chromosome A03 and another on A06, was most likely. Permutation-based testing of an assumed two-QTL model reinforced this suggestion; the only pairs of marker loci to pass statistical significance thresholds were those loci already identified by QTL modeling as part of an additive model without epistatic interaction. Both IM and CIM consolidated this result with significant LOD scores determined for markers across chromosomes A03 and A06 (Figure 4). Haley-Knott, marker regression and expectation-maximization QTL mapping algorithms produced highly analogous results with QTL intervals of 12.6 and 27.3 cM on chromosomes A03 and A06, respectively.

#### TWBJ14 GBS

Results using TWBJ14 BC_1_ GBS data were comparable to those derived from the 90K SNP array. QTL modeling of the GBS data suggested putative QTLs on chromosomes A03 and A06 with equivalent permutation-based results to those of the SNP array data. The most refined TWBJ14 QTL intervals from GBS data when expanded to interval-flanking markers were similar to those identified using the 90K SNP array. GBS defined QTL intervals for TuMV resistance of 10.4 cM on chromosome A03 and 11.9 cM on A06.

The distinct TWBJ14 GBS and SNP array-derived linkage maps could not be directly compared, although the translation of QTL-flanking markers to the *B. juncea* reference assembly suggested the QTL results from each genotyping method were very similar. For chromosome A03, the physical QTL intervals derived from GBS and SNP array data spanned 4.86 and 5.54 Mb, respectively. The lower boundaries of these intervals were predicted to be near-identical (<2 Kb apart) with a difference between upper physical QTL interval boundaries of 0.68 Mb. A physical QTL interval of 10.8 Mb on A06 was predicted from GBS data.

Too few TuMV-susceptible (31/+; 30/+_N_) samples were available for GBS to facilitate effective marker screening and linkage map production for the additional necrotic phenotype.

#### TWBJ14 Necrotic Phenotype

**Figure 5:**
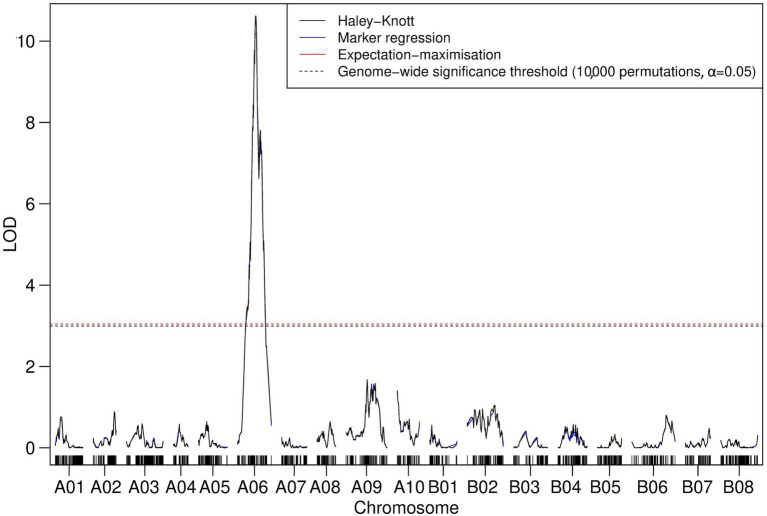
Logarithm of the odds profile of nonparametric/binary IM performed on TuMV-susceptible plants from the TWBJ14 *B. juncea* BC_1_ TuMV mapping population. A model of one QTL associated with the necrotic phenotype is assumed. A single QTL for the necrotic phenotype linked with TuMV infection on chromosome A06 passed all QTL mapping algorithm-specific thresholds for significance (dashed lines; based on an alpha significance value of 0.05 and 10,000 permutations).

The presence of three phenotypes (Figure 1) across the TWBJ14 BC_1_ population facilitated additional analyses using 90K SNP array data aimed at mapping the necrotic phenotype (+_N_). Interval mapping and CIM on the subset of 166 TuMV-susceptible BC_1_ individuals (105/+; 61/+_N_) implied a single highly significant QTL on chromosome A06 was present with an interval of 6.8 cM (Figure 5). No pairs of QTLs, full or additive models were identified which met permutation-based significance thresholds for a non-single QTL model. The physical QTL interval was determined as 2.17 Mb on chromosome A06, within the 10.8 Mb interval suggested from GBS data where resistance was explored.

#### TWBJ20 Source of TuMV Resistance

**Figure 6:**
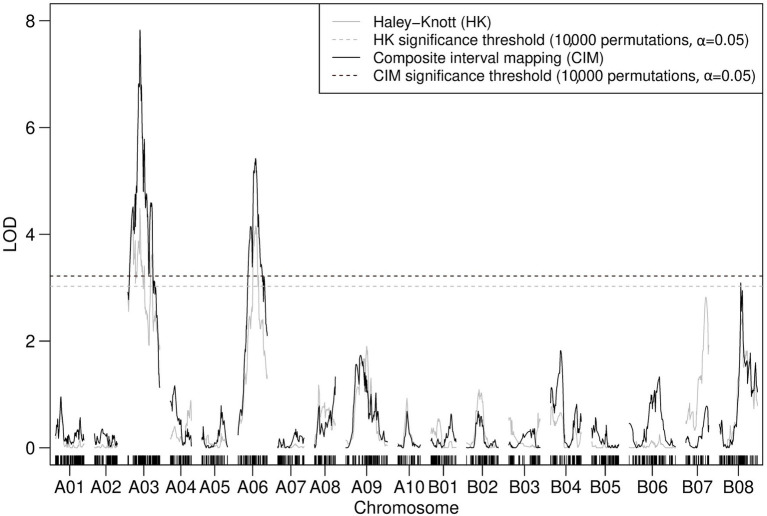
Logarithm of the odds profile of nonparametric CIM with two cofactors (assigned to the peak LOD scores for A03 and A06 determined by IM) performed on the TWBJ20 *B. juncea* BC_1_ TuMV resistance mapping population using 90K SNP array data. The TuMV resistance QTLs on A03 (*retr06*) and on A06 (*retr04*) passed the threshold (black-dashed line) for significance (LOD 3.39; based on an alpha significance value of 0.05 and 10,000 permutations). The results and significance threshold of nonparametric IM, assuming a single QTL model, using the Haley-Knot algorithm (gray) are also presented.

Composite interval mapping with two cofactors assigned to linkage map loci of peak LOD scores identified through prior IM suggested the presence of two significant QTLs associated with the resistance to TuMV in TWBJ20 (Figure 6). Modeling for QTLs using the TWBJ20 SNP array data suggested that the inclusion of three loci, one on A03, A06 and B08 chromosomes, was the most likely. This three-QTL model could not be assessed for statistical significance, however, and the addition of chromosome B08 was considered to be perhaps erroneous, related to an increased rate of recombination recorded across B08. Composite interval mapping with additional cofactors assigned incrementally across peak LOD score-associated linkage map loci did not identify any putative QTLs passing CIM-specific significance thresholds, except for those on chromosomes A03 and A06 (Figure 6). Assuming a two-QTL model, the only pair of marker loci shown to be significant for a full or additive model were identical to those identified by QTL modeling, excluding the suggested addition of a QTL on chromosome B08. The most refined QTL intervals when expanded to interval-flanking markers for chromosomes A03 were calculated as 8.9 and 21.9 cM for A06. Translation of these intervals to physical marker loci suggested a large 17.3 Mb region on chromosome A06, spanning the proposed QTL interval associated with TWBJ14 TuMV resistance, and a smaller 3.0 Mb region on chromosome A03 distinct from the TWBJ14 A03 QTL.

### TWBJ14 and TWBJ20 Complementation Testing

A slight disparity was observed in TuMV susceptibility during resistance complementation testing of F_2_ crosses depending upon whether TuMV-resistant TWBJ14 or TWBJ20 S_2_ plants were paternal or maternal parents of two F_1_ populations used for F_2_ production. The F_2_ population produced from germplasm, where the maternal F_1_ parent was a TWBJ14 S_2_ plant exhibited clear segregation for TuMV resistance with visual phenotypes correlating to PTA-ELISA data (Table 5). A contrasting less distinct segregation of TuMV resistance was recorded, where TWBJ20 material was the maternal F_1_ parent.

**Table 5:** Response of F_2_ populations where TuMV-resistant *B. juncea* TWBJ14 and TWBJ20 S_2_ plants were paternal or maternal plants of two F_1_ populations used for F_2_ production.

Maternal parent of F_1_ germplasm used for F_2_ generation (population)	No. F_2_ plants (phenotype^*1^)		Assumed number of shared TuMV resistance-associated loci	Predicted ratio for TuMV resistance:susceptibility	Goodness of fit/*χ*^2^ value^*2^
	Resistant (0)	Susceptible (+)			
TWBJ14 (S_2_)	41	51	0	31:225	91.1
TWBJ14 (S_2_)	41	51	1	7:9	0.025
TWBJ20 (S_2_)	44	39	0	31:225	118.6
TWBJ20 (S_2_)	44	39	1	7:9	3.49

*1 Phenotypes were scored according to the system of Jenner and Walsh (1996). 0, resistance with no TuMV-associated symptoms or +, systemic TuMV infection. TuMV detection using plate-trapped antigen PTA-ELISA confirmed the resistance/susceptibility status of all plants.

*2 Goodness of fit based on chi-square (*χ*^2^) method; with one degree of freedom: *χ*^2^_0.05_ = 3.84, *χ*^2^_0.01_ = 6.64, and *χ*^2^_0.001_ = 10.83.

Irrespective of F_1_ parental maternity/paternity, segregation for resistance recorded for F_2_ populations suggested that one TuMV resistance-associated locus was shared between TWBJ14 and TWBJ20. Where TWBJ14 germplasm represented the maternal F_1_ parent, the resultant F_2_ population segregated for TuMV resistance (41 resistant/0; 51 susceptible/+); this was determined by chi-squared (*χ*^2^) analysis to not be significantly different from the ratio of 7 resistant: 9 susceptible expected, where one resistant locus was shared (*χ*^2^ = 0.025, value of *p* > 0.05) and differing significantly (*χ*^2^ = 91.1, value of *p* < 0.001), where no resistance loci were in common (predicted segregation ratio of 31 resistant/0: 225 susceptible/+). Where TWBJ20 germplasm represented the maternal F_1_ parent, respective *χ*^2^ values of 3.49 (value of *p* > 0.05) and 118.6 (value of *p* < 0.001) were determined, confirming that one resistance locus was shared between the two *B. juncea* lines (Table 5).

## Discussion

### TWBJ14 and TWBJ20 Resistances to Turnip Mosaic Virus

In this study, we report the characterization of two new sources of broad-spectrum, recessive resistance effective against representative isolates of the most common pathotypes (1, 3 and 4) of TuMV. Both resistances involve two recessive genes. Resistance complementation testing and QTL analyses showed that the resistance-associated locus on chromosome A06 (*retr04*) is shared between TWBJ14 and TWBJ20, *retr05* is specific to the former and *retr06* is specific to the latter.

To date, at least 20 TuMV resistance-associated genes/loci have been mapped across all species of the *Brassica* genus (Li et al., 2019; Palukaitis and Kim, 2021) with significant efforts to characterize some (Walsh et al., 1999; Jenner et al., 2002a; Hughes et al., 2003; Qian et al., 2013; Nellist et al., 2014; Shopan et al., 2017). No sources of TuMV resistance across any *Brassica* species have been mapped to the “B” genome, and a single weak quantitative resistance to one TuMV isolate has been identified and mapped on the “C” genome (Walsh et al., 1999). Due to the evolutionary history of the *Brassica* genus, valuable traits of interest already identified in diploid *Brassica* genomes have the potential to be exploited in amphidiploid *Brassica* species through modern genetic editing techniques, or *via* interspecific crossing/resynthesis. This approach was used to great effect when introducing clubroot disease resistance from *B. rapa* into *B. juncea* material, wherein natural resistance is not evident (Hasan and Rahman, 2018) and to introduce turnip yellows virus resistance from *Brassica oleracea* and *B. rapa* into *B. napus* (Greer et al., 2021). Potential therefore exists for the generation of enhanced TuMV-resistant *B. juncea* cultivars using resistances already identified on the “A” genome of other *Brassica* species *via* resynthesis. Deployment of resistances in such a manner is, however, more difficult and time-consuming compared to the use of traditional MAS during natural introgression of any resistance-associated gene(s) from similar *B. juncea* material.

Few sources of TuMV resistance capable of being deployed directly through MAS have been identified within *B. juncea* germplasm (Nyalugwe et al., 2015; Shopan et al., 2017). The TWBJ14 and TWBJ20 *B. juncea* TuMV resistances identified and characterized here are consequently of high value. The vast majority of current resistances to TuMV in brassicas are also dominant and specific to discrete strains/pathotypes of TuMV. Such sources of genetic resistance to plant viruses remain valuable, but have potential to be rapidly overcome by less prevalent resistance-breaking viral mutants/recombinants (Stuthman et al., 2007). This can render such resistances ineffective if deployed irresponsibly owing to shifts in natural viral strain prevalence across a region, as is considered increasingly possible due to changes in pest and pathogen distribution predicted to occur with increasing worldwide temperatures (Bebber et al., 2013). The three recessive QTLs identified in this study appear to provide broad-spectrum resistance, as has also been shown for the *retr01* recessive resistance in *B. rapa* (Walsh et al., 2002). If QTLs identified here are shown to continue to be effective against the most prevalent TuMV pathotypes, 1, 3 and 4, then germplasm and molecular markers explored here represent valuable breeding resources for developing enhanced *B. juncea* cultivars with broad-spectrum TuMV resistance.

Segregation ratios for TuMV resistance in F_1_, F_2_ and BC_1_ TWBJ14 and TWBJ20 populations established that all sources of resistance investigated here were recessive. As *retr04* was mapped to chromosome A06 and *retr05* and *retr06* were both mapped to chromosome A03, and the only other mapped recessive resistances to TuMV in brassicas have been mapped to chromosome A04 (*retr01*/*retr02*; Nellist et al., 2014) and chromosome A01 (*retr03*; Shopan et al., 2017), it is clear the resistances reported here are new. It is currently unknown how these new resistances to TuMV may vary in instances where single resistance-associated loci are present.

Sequence information used to produce the 90K SNP array markers that flanked the highly significant QTL for the necrotic phenotype on chromosome A06 was homologous to specific A06 loci of a reliable *Brassica* A genome reference assembly (Belser et al., 2018). This enabled comparison to published TuMV resistance genes. The dominant resistance gene *TuRB01* and its likely ortholog *TuRB01b* were both mapped to chromosome A06 (Walsh et al., 1999; Lydiate et al., 2014). Further evidence was provided to indicate that these genes are similar or identical alleles and that *B. napus* may have acquired *TuRB01* from the *B. rapa* gene pool during *Brassica* speciation (Walsh et al., 2002; Lydiate et al., 2014). Unpublished work undertaken by collaborators refined candidates of *TuRB01*/*TuRB01b* to a bacterial artificial chromosome contig. The full sequence of this contig aligned to chromosome A06 of the Belser et al. (2018) reference genome with 99.8% homology. This region was contained within the A06 necrotic phenotype QTL interval determined here. This could suggest the presence of *TuRB01b* in the plant lines investigated here, originating from a natural hybridization between a *B. rapa* plant possessing *TuRB01b* and *B. nigra*.

In terms of the TuMV resistances identified in this study, reliable identification of candidate genes for *retr04*, *retr05*, and *retr06* was not possible with currently available data. This was due to the large number of genes suggested within QTL intervals and because robust gene annotation/validation has not been performed on the unpublished *B. juncea* reference assembly used in this study. Previously identified recessive TuMV resistance genes *retr01*, *retr02* and *retr03* have all been shown as variants of components of the eukaryotic translation initiation complex (Nellist et al., 2014; Shopan et al., 2017). Most recessive plant resistances to potyviruses are due to natural mutations in *eukaryotic translation initiation factor 4E* (*eIF4E*) genes (Robaglia and Caranta, 2006). As with *retr01*-based resistance in *B. rapa*, it is possible that *eIF4E/eIF(iso)4E* allelic variants (which TuMV cannot use) could be candidates for *retr04*, *retr05*, and *retr06*. Shopan et al. (2020) used sequence data of 95 established *eukaryotic translation initiation factors* (*eIFs*) from *Arabidopsis thaliana* to identify homologs across various *Brassica* species, including 190 putative *B. juncea eIFs* (*BjueIFs*). Using the positions of these *BjueIFs* on the *B. juncea* cv. Tumida assembly developed by Yang et al. (2016) and alignment of the *retr04*, *retr05*, and *retr06* QTL-flanking SNP array markers, *BjueIFs* within all QTLs were identified (Supplementary Figure 1).

The only *BjueIFs* suggested in the chromosome A06 QTL associated with TWBJ20 were *BjuA022587* and *BjuA023685*; these are both proposed by Shopan et al. (2020) to be homologs of *eIF2ß*. Of these two putative *eIF2ß* genes, *BjuA023685* was also contained within the A06 QTL associated with TWBJ14 resistance. *BjuA023685* may perhaps represents a candidate for *retr04* shared by TWBJ14 and TWBJ20. Five additional *BjueIFs* (putative homologs of *eIF2C*, *eIF3*, *eIF3δ*, *eIF4A*, *eIF4B*, and *eukaryotic release factor*, *RF*) were also contained in the TWBJ14 A06 QTL (Supplementary Figure 1). Within the first A03 *retr05*/TWBJ14-associated QTL, six putative *BjueIFs* are suggested, all of which are subunits of *eIF3*, *eIF4*, or an *eIF4A*-like RNA helicase. Only two *BjueIFs* (*BjuA010155*; *eIF4A* and *BjuA010508*; *eIF3F*) were located within the *retr06*/TWBJ20 A03 QTL. While *BjueIFs* suggested in these QTLs may represent candidates for *retr04*, *retr05*, and *retr06*, the *B. juncea* cv. Tumida genome assembly employed by Shopan et al. (2020) may not accurately represent e*IF* variation in germplasm explored here. Future analyses of putative e*IF*s using the unpublished *B. juncea* reference assembly based on the specific 060DH17 line used in this study may elucidate if these e*IF*s are likely candidate resistance genes of interest. Molecular markers/polymorphic loci identified here could also be investigated against second backcross (BC_2_) populations as part of work intending to refine candidate gene intervals, whilst validating prospective markers for commercial use. Additional characterization of the TWBJ14 and TWBJ20 resistances is required, but these new resistances present promise as a genetic resource for helping to mitigate TuMV.

### Processing of Recalcitrant Genotyping by Sequencing Data

For reasons comprehensively reviewed previously (Davey et al., 2011; Deschamps et al., 2012; Poland and Rife, 2012; Kim et al., 2016), GBS is considered to be an informative, reliable, and highly versatile genotyping option. In circumstances where an appropriate or reliable reference genome is unavailable, the massive high-throughput parallel sequencing associated with NGS-based techniques may represent one of few viable options for identifying genome-wide SNP/InDels in original germplasm. The use of GBS here facilitated the development of a reasonably robust linkage map using the most informative subset of 481 TWBJ14 BC_1_-specific markers. The unfiltered data used to identify this subset presented significant issues, many of which are commonplace for GBS. Data produced by GBS tends to contain large quantities of missing genotyping calls compared to alternative methods, whilst also exhibiting inherent and often considerable error rates (Furuta et al., 2017). Missing GBS data can be attributed to presence/absence variation in germplasm under investigation, polymorphic restriction sites impacting the generation of reduced representation libraries, and/or differential methylation across DNA (Poland and Rife, 2012). Technical issues that contribute to missing data include the complexity of reduced genome representation libraries, number of unique sequence tags and overall sequence coverage. These issues may have occurred during employment of GBS here.

Sequencing read depth/coverage is the predominant measure used for rapid screening of NGS-based data for marker quality. As a case study, the processing of GBS data here represents a situation not previously reported. Highly recalcitrant genotypic data has been used to produce results comparable with those derived from near-optimal 90K SNP array data without any implementation of coverage-based screening of prospective markers. The consideration that this was achieved, where TWBJ14 90K SNP array data was generated from more than double the number of GBS BC_1_ samples rendered this output increasingly striking. Read coverage cannot be considered an absolute indication of reliability nor measure for whether potential marker loci may ultimately prove informative. This was demonstrated here wherein “traditional” read depth-based screening of TWBJ14 GBS data proved unviable for producing a marker subset for effective linkage map production. Based on any filters applied according to sequencing coverage, a large quantity of GBS marker loci were considered reliable yet proved detrimental for effective linkage map production. The majority of these “reliable” marker loci presented qualitatively impossible genotyping because of the backcrossing strategy employed and allelic variation observed across BC_1_ samples.

Other methods including the imputation-based methods of the “ABHgenotypeR” R package (Furuta et al., 2017) and hidden Markov models (Technow and Gerke, 2017) proved ineffective for processing the imperfect GBS data explored here. In contrast to using imputation-based methods, all questionable GBS marker loci identified here were removed without any amendments whatsoever made to original data. The methods implemented here limited prospective markers available for linkage map production and QTL analyses, although this was considered to not have significant impact due to the limited levels of recombination observed across the 102 TWBJ14 BC_1_ plant samples assessed. Reductive marker screening approaches are speculated to be advantageous relative to strategies reliant on data amendment. A key advantage of this reductive approach relative to imputation is the removal of erroneous QTLs caused by false remediation of genotyping calls implemented because of cumulative genotyping errors or sparsity of markers.

It is debatable whether GBS is ever an appropriate method to employ for trait-mapping studies involving a large number of mapping population samples, due to both cost-efficiency and data handling considerations. Genotyping by sequencing routinely generates a large quantity of potentially useful markers. A level of diminishing returns can exist, however, for studies reliant on biparental mapping populations. The addition of markers where such a population has reached a stage of marker saturation with all recombination events captured can be considered detrimental, does not provide increased QTL interval resolution and may convolute analyses (Poland and Rife, 2012). This was likely the case for GBS performed here. Irrespective of the 47,848 marker loci initially suggested as perhaps informative prior to any screening for marker quality, captured recombination ultimately proved a limiting factor for whether markers were informative. During the final GBS marker loci filtering stage, wherein any loci demonstrating near-identical recombination fraction were removed, the reliable 1,057 marker subset was reduced to the representative 481 used for QTL analyses (Supplementary Table 2). The many benefits of GBS for QTL mapping, including no prior information of a target species genome being required and the ability to identify germplasm-specific markers, mean that such methods are highly valuable (Kim et al., 2016). This is regardless of whether recombination represents a bottleneck for identifying informative markers. Ultimately, the use of similar reductive screening methods to those employed here without read coverage being implemented as a predominant measure of marker quality may prove effective for other similar biparental QTL mapping studies.

## Data Availability Statement

The original contributions presented in the study are included in the article/**Supplementary Material**, further inquiries can be directed to the corresponding authors.

## Author Contributions

JAW, GCB, and TW planned the genetic analysis and mapping population development. IAPP and LEB conceived the TuMV resistance mapping. JAW and LEB designed the genetic complementation experiment and wrote the manuscript. LEB and TW conducted the experiments. EEH and IAPP genotyped the BC_1_ populations. LEB constructed genetic linkage maps and carried out QTL analysis on the BC_1_ populations. All authors contributed to the article and approved the submitted version.

## Funding

Research in this manuscript was part of LEB’s PhD which was funded by a UK Biotechnology and Biological Sciences Research Council (BBSRC) industrial Collaborative Awards in Science and Engineering (iCASE) studentship with Sakata UK Ltd. (BB/M016447/1) and TW’s PhD which was funded by the University of Warwick and the China Scholarship Council. Funding from BBSRC’s Newton Fund Pulses and Oilseeds Research Initiative to JAW (BB/R019819/1) is also acknowledged. The AAFC Canadian Crop Genomics Initiative is also acknowledged for funding associated with IAPP’s and EEH’s contributions. The Open Access publication costs were provided by the UKRI block grant to the University of Warwick.

## Conflict of Interest

The authors declare that the research was conducted in the absence of any commercial or financial relationships that could be construed as a potential conflict of interest.

The handling editor declared a past co-authorship with some of the authors (JAW and GCB).

## Publisher’s Note

All claims expressed in this article are solely those of the authors and do not necessarily represent those of their affiliated organizations, or those of the publisher, the editors and the reviewers. Any product that may be evaluated in this article, or claim that may be made by its manufacturer, is not guaranteed or endorsed by the publisher.

## Supplementary Material

Supplementary Figure 1 Chromosomes A03 and A06 of the *Brassica juncea* cv. Tumida reference assembly with TWBJ14 (black) and TWBJ20 (red) turnip mosaic virus resistance-associated QTLs presented (based on sequence alignment of QTL-flanking SNP array markers). Also presented are loci associated with putative *Arabidopsis thaliana eukaryotic translation initiation factor* homologs in *B. juncea*.
