# Supplementary material for: Characterization and Mapping of retr04, retr05 and retr06 Broad-Spectrum Resistances to Turnip Mosaic Virus in Brassica juncea, and the Development of Robust Methods for Utilizing Recalcitrant Genotyping Data
Source: Front Plant Sci. 2022 Jan 12;12:787354. doi: 10.3389/fpls.2021.787354 (PMC8790578; doi:10.3389/fpls.2021.787354)
Supplement: Supplementary Figure 1 — Chromosomes A03 and A06 of the Brassica juncea cv. Tumida reference assembly with TWBJ14 (black) and TWBJ20 (red) turnip mosaic virus resistance-associated QTLs presented (based on sequence alignment of QTL-flanking SNP array markers). Also presented are loci associated with putative Arabidopsis thaliana eukaryotic translation initiation factor homologs in B. juncea. [file Data_Sheet_1.zip › Supplementary Table 1 and 2.docx]

***Supplementary Material***

Supplementary Table 1. Successive reductive filters applied to 90K SNP array genotyping data of both *Brassica juncea* TWBJ14 and TWBJ20 BC_1_ turnip mosaic virus resistance mapping populations, and the associated impacts upon the number of retained marker loci.

| Filter stage | Filter of genotyped loci or, where stated, BC_1_ samples removed | Number of genotyped marker loci retained after each filter stage | |
| --- | --- | --- | --- |
|  |  | TWBJ14 BC_1_ | TWBJ20 BC_1_ |
| 0 | N/A | 11,732 | 11,732 |
| 1 | 060DH17 genotyping identified as heterozygous | 9,829 | 9,829 |
| 2 | Monomorphic across BC_1_ samples | 8,387 | 8,227 |
| 3 | Genotypic data recorded in fewer than 75% of BC_1_ samples | 8,241 | 8,007 |
| 4 | BC_1_ samples removed where fewer than 75% of remaining marker loci were genotyped | 8,241  (no samples removed) | 8,007  (no samples removed) |
| 5 | Genotypic data recorded in fewer than 95% of samples | 7,349 | 7,420 |
| 6 | Allele frequency bias away from a 1:1 ratio (p-value < 10 x 10^-10^) observed across BC_1_ samples | 7,088 | 7,196 |
| 7 | Not assigned to 18 largest linkage groups, based on calculated pairwise recombination fractions | 7,087 | 7,184 |
| 8 | Matching pattern of genotyping observed across BC_1_ samples | 1,696 | 3,227 |
| 9 | Genotypic data recorded in fewer than 95% of samples | 1,671 | 3,167 |
| 10 | Misassigned to chromosomes/linkage groups (based on *B. juncea* reference genome assembly) | 1,663 | 3,158 |
| 11 | Recombination fraction calculated as near-identical (all except one removed; threshold < 0.00001 RF difference) | 1,064 | 966 |

Supplementary Table 2. Successive reductive filters applied to GBS-derived genotyping data of *Brassica juncea* TWBJ14 BC_1_ turnip mosaic virus resistance mapping populations, and the associated impact upon the number of retained marker loci.

| Filter stage | Filter of genotyped loci or, where stated, BC_1_ samples removed | Number of genotyped marker loci retained after each filter stage |
| --- | --- | --- |
| 0 | N/A | 47,845 |
| 1 | Genotypic data recorded in fewer than 75% of BC_1_ samples | 10,798 |
| 2 | BC_1_ samples removed where fewer than 50% of remaining marker loci were genotyped | 10,798  (1 sample removed) |
| 3 | Genotypic data recorded in fewer than 90% of BC_1_ samples | 8,282 |
| 4 | Allele frequency bias away from a 1:1 ratio (p-value < 10 x 10^-10^) observed across BC_1_ samples | 7,088 |
| 5 | Not assigned to 18 largest linkage groups, based on calculated pairwise recombination fractions | 2,690 |
| 6 | Matching pattern of genotyping observed across BC_1_ samples | 1,358 |
| 7 | Genotypic data recorded in fewer than 90% of BC_1_ samples | 1,063 |
| 8 | Misassigned to chromosomes/linkage groups (based on *B. juncea* reference genome assembly) | 1,057 |
| 9 | Recombination fraction calculated as near-identical (all except one removed; threshold < 0.00001 RF difference) | 481 |
