# Supplementary figures and images for: Characterization and Mapping of retr04, retr05 and retr06 Broad-Spectrum Resistances to Turnip Mosaic Virus in Brassica juncea, and the Development of Robust Methods for Utilizing Recalcitrant Genotyping Data
Source: Front Plant Sci. 2022 Jan 12;12:787354. doi: 10.3389/fpls.2021.787354 (PMC8790578; doi:10.3389/fpls.2021.787354)

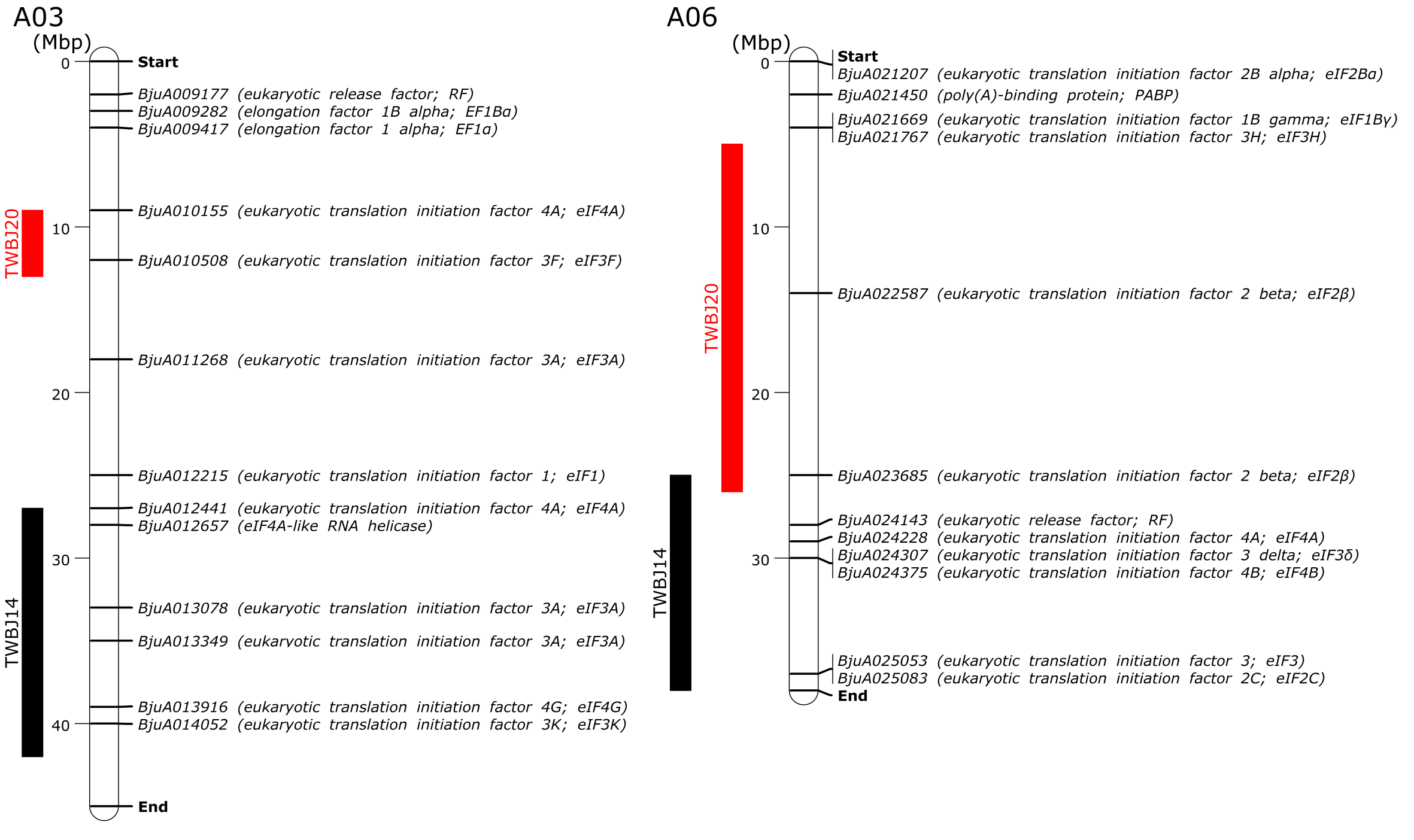

Supplement: Supplementary Figure 1 — Chromosomes A03 and A06 of the Brassica juncea cv. Tumida reference assembly with TWBJ14 (black) and TWBJ20 (red) turnip mosaic virus resistance-associated QTLs presented (based on sequence alignment of QTL-flanking SNP array markers). Also presented are loci associated with putative Arabidopsis thaliana eukaryotic translation initiation factor homologs in B. juncea. [file Data_Sheet_1.zip › Supplementary Figure 1.TIFF]
